# The effect of coffee and black tea consumption on sleep bruxism intensity based on polysomnographic examination

**DOI:** 10.1016/j.heliyon.2023.e16212

**Published:** 2023-05-12

**Authors:** Weronika Frosztega, Mieszko Wieckiewicz, Dorian Nowacki, Rafal Poreba, Gabriella Lachowicz, Grzegorz Mazur, Helena Martynowicz

**Affiliations:** a Department of Internal Medicine, Occupational Diseases, Hypertension and Clinical Oncology, Wroclaw Medical University, 213 Borowska St., 50-556 Wroclaw, Poland; b Student Research Club No K133, Faculty of Medicine, Wroclaw Medical University, 50-367 Wroclaw, Poland; c Department of Experimental Dentistry, Wroclaw Medical University, 26 Krakowska St., 50- 425 Wroclaw, Poland; d Department of Human Nutrition, Wroclaw University of Environmental and Life Sciences, 37 Chelmonskiego St., 51-630 Wroclaw, Poland

**Keywords:** Sleep bruxism, Coffee, Black tea, Caffeine, Arousal, Sleep architecture, Uric acid

## Abstract

**Background:**

Sleep bruxism (SB) is a common behavior that can result in various clinical consequences on human health. Risk factors for SB include among others emotional stress, anxiety, tobacco smoking, and excessive alcohol consumption. Coffee and black tea are among the most commonly consumed beverages worldwide. This study explores the influence of coffee and black tea consumption on bruxism intensity, as observed in polysomnographic examination.

**Methods:**

Polysomnographic examination with simultaneous camera recording was conducted in 106 adult subjects. The results were evaluated according to guidelines set out by the American Academy of Sleep Medicine (AASM). The study group was divided according to habitual stimulant usage, as declared by the participants in a self-reported questionnaire. Four groups were identified: coffee drinkers versus non-drinkers and black tea drinkers versus non-drinkers.

**Results:**

The bruxism episode index (BEI) was increased in coffee-drinkers as opposed to non-drinkers (4.59 ± 3.44 vs. 2.87 ± 1.50, p = 0.011). Sleep fragmentation, measured according to the arousal index, was comparable in coffee drinkers and non-drinkers. Electrolyte and lipid levels were similar in coffee drinkers and non-drinkers. Habitual black tea intake did not affect sleep architecture or bruxism intensity.

**Conclusions:**

The study showed that habitual coffee consumption is a risk factor for the increased intensity of sleep bruxism. Neither coffee nor tea consumption is related to sleep fragmentation in habitual drinkers. Coffee and tea intake does not affect electrolyte and lipid concentrations. Caution should therefore be recommended in drinking coffee in people with sleep bruxism.

## 1 Introduction

Various definitions of bruxism have been discussed over a number of decades. The term “la bruxomanie” was first coined at the beginning of the twentieth century by Marie Pietkiewicz [1]. In 2013 Lobbezoo et al. defined sleep bruxism (SB) as a “repetitive jaw-muscle activity characterized by clenching or grinding of the teeth and/or bracing or thrusting of the mandible” [2]. In 2018 Lobbezoo et al. updated the definition, distinguishing between two separate definitions for awake bruxism and sleep bruxism [3]. Rather than being referred to as a disorder, the term bruxism was now defined as a behavior at risk of certain clinical consequences in healthy individuals. Moreover, diagnostic techniques for sleep bruxism were divided into instrumental (polysomnography (PSG), as a gold standard tool in bruxism diagnosis in sleep laboratory and surface electromyography with or without heart rate (HR) recording at home) and non-instrumental (self-reports and clinical inspection findings) [3] Sleep bruxism is a common phenomenon worldwide, with a prevalence of 8–13% in the general population [4]. Risk factors for SB have previously been examined and include (among others), emotional stress, tobacco smoking, excessive alcohol consumption, sleep apnea and anxiety disorders [5]. Clinical manifestations of SB may include tooth wear, masticatory muscle pain, and headache [6,7].

Coffee and black tea are some of the most frequently consumed beverages worldwide. The consumption patterns vary between countries, due to cultural traditions/backgrounds and habits ingrained in society. Norway is the leader in per capita coffee consumption in Europe [8] It has been established, that drinking caffeinated beverages has gained in popularity over the last decade and about 85% of the U.S. population drinks one or more caffeinated beverages a day [9]. Furthermore, the last few years have been dominated by the ongoing COVID-19 pandemic, which has also left its mark on dietary behaviors, including caffeine consumption [10].

The two most prominent sources of caffeine are coffee and black tea [11]. Caffeine, namely 1,3,7-trimethylxanthine, is a purine alkaloid occurring naturally in coffee beans [12]. Caffeine is rapidly absorbed, has a high bioavailability and has biological effects on the human body via its blockage of the widely distributed adenosine receptors (A1 and A2A). Ultimately it leads to the stimulation of the respiratory, renal, cardiovascular, gastrointestinal and central nervous systems, as well as adipose tissue [[13], [14], [15]]. Adenosine in general promotes sleep. Due to the blockage of these receptors, caffeine intake acts as a stimulus and promotes wakefulness/has a positive influence on fatigue, reaction time, enhances attention and improves athletic performance [[16], [17], [18]]. It has been reported that the risk of developing Parkinson's disease, dementia, Alzheimer disease, type 2 diabetes, chronic liver cirrhosis as well as apnea in premature infants is significantly reduced with caffeine consumption [15]. Caffeine (≥100 mg) is also widely used as a co-analgetic, increasing analgesic potential in pain management [19].

While the standard size of a cup of coffee may vary, it is estimated that a single cup of coffee provides about 100 mg of caffeine, while a cup of black tea contains about 55 mg of caffeine [13,20]. A caffeine overdose is associated with anxiety, tremulousness and sleeping difficulties [21]. The estimated fatal dose of caffeine is about 10–14 g [16,22]. It is worth noting though, that caffeine is not only found in coffee and black tea. It also occurs naturally in various plants such as cocoa beans, guarana berries and yerba mate leaves. Artificially, it is added to energy drinks.

Besides the caffeine content, coffee is also a rich source of micronutrients including nicotinic acid, niacin, vitamin E, magnesium and potassium. The average micronutrient quantity however varies considerably depending on the coffee brewing method [13].

Caffeine has been the subject of research in the past and has been shown to be a risk factor for developing sleep bruxism. However previous studies were predominantly based on questionnaires or self-reports, and had no possibility of diagnosing definite bruxism, or assessing its intensity. Moreover, the consumption of black tea and its compound theanine, has as yet not been investigated in the context of SB. To the best of our knowledge, there are no research papers investigating the impact of coffee and tea consumption on the intensity of sleep bruxism using polysomnographic assessment with camera recording, being gold standard tool for used in SB diagnosis [3]. The aim of this study is to define the effect of habitual coffee and black tea consumption on sleep bruxism intensity and alterations in sleep architecture.

## 2 Materials and methods

### 2.1 Participants

This was a prospective observational case-control study. The study was conducted in the Sleep Laboratory at the Department and Clinic of Internal Medicine, Occupational Diseases, Hypertension and Clinical Oncology at Wroclaw Medical University. All patients were referred to the Sleep Laboratory because of a suspicion of a sleep disorder. The study was approved by the Ethics Committee at Wroclaw Medical University (approval no. KB-790/2022). All procedures were performed in accordance with the Declaration of Helsinki. Voluntary informed consent (signed) was obtained from the participants preceding the start of the study.

After fulfilling specific inclusion and exclusion criteria requirements, 106 patients were included in the study group. The inclusion criteria were as follows: age >18 years and providing written informed consent to participate in the study. Subjects were excluded from the study if they: presented neurological disorders and/or neuropathic pain, active inflammation, confirmed active malignancy, severe respiratory and cardiac insufficiency, had treatments affecting muscle function and sleep structure, had severe mental disorders or cognitive disability, were pregnant, or if there was a lack of compliance during the study.

Patients were divided into groups based on their declaration of coffee and black tea consumption respectively. Four groups of patients were identified: coffee drinkers versus coffee non-drinkers and black tea drinkers versus black tea non-drinkers.

At admission, patients completed the self-reported questionnaire. Questions about coffee and black tea consumption were evaluated. The questionnaire included a question about the number of cups of coffee or black tea that was consumed on average in a day (between 1 and 10 cups).

**Fig. 1:**
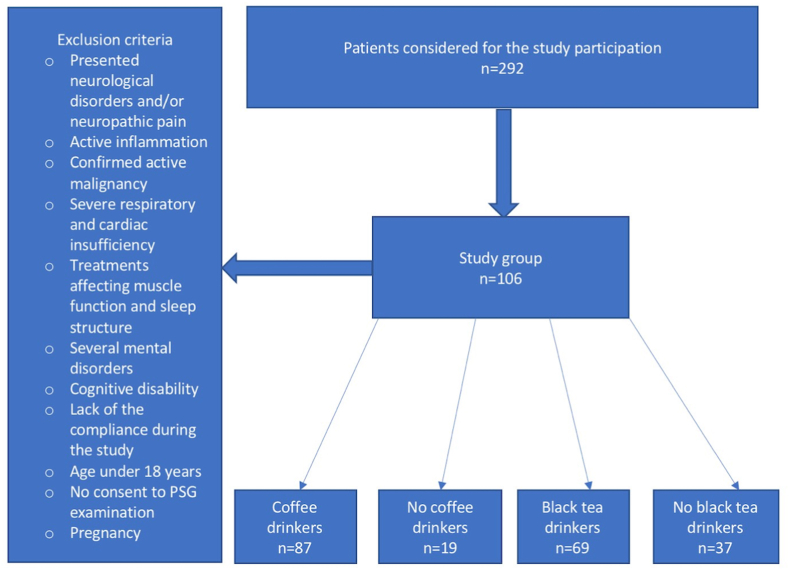
Flowchart presenting the study design.

The sample size was calculated using an online calculator (https://www.calculator.net/sample-size-calculator.html). With a margin of error of 5% and confidence level of 95%, the minimal sample size was set at 80. With a study group consisting of 106 subjects, the required criterion had been met. Fig. 1 presents a flowchart of the study design.

### 2.2 Polysomnography

Patients underwent a previously prescribed polysomnographic (PSG) examination. Video polysomnography was performed with a Nox A1 (NOX Medical, Reykjavík, Iceland) device. Patients were recorded between 22:00 and 6:00, taking into account the wishes and habits of the patient, in order to maintain the natural circadian rhythm. Electromyographic electrodes were attached as recommended by the American Academy of Sleep Medicine (AASM) guidelines [23]. The examination included audio and video recordings together with electrocardiographic, electroencephalographic, electrooculographic, electromyographic recordings, body position detection and thoracic and abdominal breathing activity. A NONIN WristOx2 3150 pulse oximeter (Nonin Medical Inc., Plymouth, MN, USA) was used to measure saturation level (SpO2%), pulse, and plethysmography data. Breathing, sleep bruxism (SB) and sleep parameters were collected. Sleep parameters were assessed and classified according to standard AASM criteria [23]: non-REM 1 sleep stage (N1), non-REM 2 sleep stage (N2), non-REM 3 sleep stage (N3), REM sleep stage, arousal index (AI), sleep latency (SL), sleep efficiency (SE), apnea-hypopnea index (AHI), periodic limb movement disorder (PLMD), oxygen desaturation index (ODI) and snore. SB parameters were assessed: bruxism episode index (BEI), phasic bruxism, tonic bruxism, and mixed bruxism. The classification of sleep bruxism was based on the number of bruxism episodes per hour of sleep (BEI) and assigned as irrelevant (BEI <2), mild to moderate (BEI 2–4) and severe (BEI >4) [24].

At 7 a.m., after the conclusion of the PSG examination, blood parameters were obtained by venipuncture preceded by 12 h of overnight fasting. Blood samples were analyzed at the Main Laboratory at Wroclaw Medical University. Laboratory tests were performed according to the standard laboratory protocols at Wroclaw University Teaching Hospital.

### 2.3 Statistics

The database, gathered from 106 individuals that met the inclusion criteria, was analyzed with Statistica 13.3 (Statsoft, Poland). Variables are presented as a mean and standard deviation. First, the data was characterized by the analysis of the distribution and the variance equality. For variables that had met the parametric criteria, classic parametric analyses were used for the evaluation of the difference between two groups for independent variables (T-test). In case data had not met the criteria of a standard parametric test, nonparametric, relevant alternatives were utilised for independent samples (such as: Mann–Whitney *U* test or two sample Kolmogorov–Smirnov test). The method of data analysis was determined by meeting the statistical criteria for the relevant test. Statistical significance was recognized for a p-value <0.05.

## 3 Results

Total of 292 patients were admitted to the sleep laboratory between May 2020 and December 2021. Among them, total of 106 adults were included in the study group. The average age of the study group was 48.18 ± 16.26 years. 44% of the participants were female (n = 47), and 56% were male (n = 59). Comorbidities observed in the study group included myocardial infarction (MI), stroke, arterial hypertension, diabetes and ischemic heart disease. Data on the prevalence of comorbidities in the study group is provided in Table 1. The polysomnographic and bruxism parameters of the entire study group are presented in Table 2.

**Table 1:** The prevalence of comorbidities in the entire study group.

Parameter	Total (*n* = 106)	Coffee drinkers (*n* = 87)	No coffee drinkers (*n* = 19)	Black tea drinkers (*n* = 69)	No black tea drinkers (*n* = 37)
Myocardial infarction	6	5	1	2	4
Stroke	5	4	1	3	2
Arterial hypertension	42	33	9	27	15
Diabetes	19	15	4	11	8
Ischemic heart disease	7	5	2	3	4

**Table 2:** The polysomnographic and sleep bruxism parameters in the entire study group (n = 106).

Parameter	Mean value ± SD
AHI (n/h)	21.10 ± 24.07
ODI (n/h)	19.30 ± 21.86
Snore (%)	21.59 ± 21.46
PLMS index (n/h)	8.13 ± 18.14
SL (min)	19.42 ± 22.97
REM latency (min)	102.112 ± 76.27
WASO (min)	62.90 ± 49.89
SE (%)	82.39 ± 11.93
N1 (% of TST)	6.52 ± 5.68
N2 (% of TST)	50.42 ± 23.82
N3 (% of TST)	26.97 ± 27.42
REM (% of TST)	22.67 ± 10.22
AI (n/h)	10.85 ± 17.25
Average Sp02 (%)	92.58 ± 4.74
Minimal Sp02 (%)	81.87 ± 8.88
Sp02 duration <90% (%)	10.61 ± 17.25
Bruxism episode index (BEI) (n/h)	4.29 ± 3.25
Phasic BEI (n/h)	2.05 ± 2.37
Tonic BEI (n/h)	1.38 ± 1.19
Mixed BEI (n/h)	0.68 ± 0.69
N1 BEI (n/h)	16.34 ± 10.61
N2 BEI (n/h)	4.79 ± 4.13
N3 BEI (n/h)	2.10 ± 1.98
REM BEI (n/h)	3.42 ± 2.37
Bruxism with arousal (n/h)	2.00 ± 1.821

AHI, apnea-hypopnea index; ODI, oxygen desaturation index; PLMS, periodic limb movements syndrome; SL, sleep latency; WASO, wake after sleep onset, SE, sleep efficiency; N1, non-rapid eye movement sleep stage 1; N2, non-rapid eye movement sleep stage 2; N3, non-rapid eye movement sleep stage 3; REM: rapid eye movement; TST, total sleep time; AI, arousal index; BEI, bruxism episode index; Sp02; oxygen blood saturation, SD; standard deviation; min, minutes; n/h, number/hour.

The sleep bruxism parameters are presented in Table 3. The bruxism episode index (BEI), BEI in N1 (non-rapid eye movement sleep stage 1), BEI in N3 (non-rapid eye movement sleep stage 3) and BEI in rapid eye movement (REM) sleep stage was increased in coffee drinkers compared to non-drinkers. There were no significant differences in bruxism parameters between black tea drinkers and non-drinkers.

**Table 3:** Sleep bruxism parameters in the studied groups based on polysomnographic examination.

Parameter	Coffee drinkers	No coffee drinkers	*p*	Black tea drinkers	No black tea drinkers	*p*
Bruxism episode index (BEI) (n/h)	4.59 ± 3.44	2.87 ± 1.50	**0.011**	3.95 ± 2.44	4.91 ± 4.34	>0.05
Phasic BEI (n/h)	2.17 ± 2.56	1.47 ± 1.05	>0.05	1.75 ± 1.59	2.59 ± 3.34	>0.05
Tonic BEI (n/h)	1.36 ± 1.09	1.44 ± 1.61	>0.05	1.33 ± 1.26	1.47 ± 1.06	>0.05
Mixed BEI (n/h)	0.68 ± 0.69	0.65 ± 0.70	>0.05	0.63 ± 0.62	0.76 ± 0.82	>0.05
N1 BEI (n/h)	17.05 ± 10.42	13.10 ± 11.14	< **0.025**	15.74 ± 9.49	17.46 ± 12.50	>0.05
N2 BEI (n/h)	5.03 ± 4.21	3.73 ± 3.67	>0.05	4.46 ± 3.14	5.41 ± 5.52	>0.05
N3 BEI (n/h)	2.29 ± 2.08	1.22 ± 1.09	**0.003**	1.78 ± 1.53	2.69 ± 2.53	>0.05
REM BEI (n/h)	3.56 ± 2.22	2.80 ± 2.98	**0.019**	3.34 ± 2.51	3.58 ± 2.13	>0.05
Bruxism with arousal (n/h)	2.05 ± 1.91	1.76 ± 1.37	>0.05	1.96 ± 1.86	2.07 ± 1.78	>0.05

BEI, bruxism episode index; N1, non-rapid eye movement sleep stage 1; N2, non-rapid eye movement sleep stage 2; N3, non-rapid eye movement sleep stage 3; REM: rapid eye movement; n/h, number/hour; statistically significant values are shown in bold (*p* < 0.05).

The polysomnographic parameters in each studied group, are presented in Table 4. N2 (non-rapid eye movement sleep stage 2) sleep stage duration was decreased in coffee drinkers compared to non-drinkers. A decreased duration of wake after sleep onset (WASO) was observed in coffee drinkers. The arousal index was similar in coffee drinkers and non-drinkers. All sleep parameters were comparable in black tea drinkers and non-drinkers.

**Table 4:** Polysomnographic evaluation of the studied groups.

Parameter	Coffee drinkers	No coffee drinkers	*p*	Black tea drinkers	No black tea drinkers	*p*
AHI (n/h)	21.30 ± 24.11	20.21 ± 24.52	>0.05	21.25 ± 22.44	20.84 ± 27.15	>0.05
ODI (n/h)	19.73 ± 22.30	17.36 ± 20.19	>0.05	19.22 ± 20.22	19.44 ± 24.87	>0.05
Snore (%)	21.92 ± 21.37	20.11 ± 22.37	>0.05	21.58 ± 22.22	21.60 ± 20.30	>0.05
PLMS index (n/h)	8.60 ± 19.61	6.00 ± 8.91	>0.05	9.23 ± 21.75	6.10 ± 8.01	>0.05
SL (min)	19.54 ± 23.68	18.87 ± 20.00	>0.05	19.99 ± 25.20	18.37 ± 18.46	>0.05
REM latency (min)	98.48 ± 69.40	118.58 ± 102.57	>0.05	100.11 ± 77.12	105.80 ± 75.60	>0.05
WASO (min)	57.94 ± 43.07	85.33 ± 70.50	**0.029**	67.41 ± 50.12	54.60 ± 49.04	>0.05
SE (%)	83.20 ± 10.63	78.71 ± 16.44	>0.05	81.39 ± 11.86	84.22 ± 11.99	>0.05
N1 (% of TST)	6.31 ± 5.62	7.43 ± 6.01	>0.05	7.26 ± 6.24	5.15 ± 4.20	>0.05
N2 (% of TST)	48.02 ± 17.90	61.30 ± 40.15	**0.011**	49.48 ± 23.82	52.14 ± 24.04	>0.05
N3 (% of TST)	27.56 ± 29.68	24.30 ± 13.28	>0.05	24.72 ± 10.19	31.10 ± 44.18	>0.05
REM (% of TST)	22.91 ± 7.29	21.59 ± 18.75	>0.05	22.61 ± 11.30	22.78 ± 8.02	>0.05
AI (n/h)	10.52 ± 16.20	12.33 ± 21.85	>0.05	10.74 ± 16.99	11.04 ± 17.95	>0.05
Average Sp02 (%)	92.51 ± 5.10	92.88 ± 2.68	>0.05	92.19 ± 5.53	93.28 ± 2.69	>0.05
Minimal Sp02 (%)	82.02 ± 9.04	81.21 ± 8.31	>0.05	81.58 ± 8.12	82.41 ± 10.22	>0.05
Sp02 duration <90% (%)	10.41 ± 16.68	11.55 ± 20.11	>0.05	11.33 ± 17.34	9.29 ± 17.24	>0.05

AHI, apnea-hypopnea index; ODI, oxygen desaturation index; PLMS, periodic limb movements syndrome; SL, sleep latency; WASO, wake after sleep onset; SE, sleep efficiency; N1, non-rapid eye movement sleep stage 1; N2, non-rapid eye movement sleep stage 2; N3, non-rapid eye movement sleep stage 3; REM: rapid eye movement; TST, total sleep time; AI, arousal index; BEI, Sp02; oxygen blood saturation, SD; standard deviation; min, minutes; n/h, number/hour; statistically significant values are shown in bold (p < 0.05).

The electrolyte, lipid and CRP concentrations remained similar in all the studied groups (Table 5).

**Table 5:** The concentration of electrolytes, CRP, lipids and serum uric acid in the studied groups.

Parameter	Coffee drinkers	No coffee drinkers	*p*	Black tea drinkers	No black tea drinkers	*p*
Mg [mmol/l]	1.94 ± 0.21	2.04 ± 0.18	>0.05	1.95 ± 0.20	1.97 ± 0.22	>0.05
Na [mmol/l]	140.10 ± 2.19	140.00 ± 1.29	>0.05	140.07 ± 2.15	140.09 ± 1.87	>0.05
K [mmol/l]	4.31 ± 0.30	4.3 ± 0.23	>0.05	4.30 ± 0.25	4.38 ± 0.35	>0.05
Ca [mmol/l]	9.28 ± 0.26	9.31 ± 0.43	>0.05	9.28 ± 0.28	9.31 ± 0.34	>0.05
Total cholesterol [mg/dl]	204.53 ± 44.66	197.16 ± 63.24	>0.05	204.62 ± 50.26	200.37 ± 45.82	>0.05
LDL [mg/dl]	118.15 ± 38.91	119.32 ± 48.57	>0.05	119.60 ± 40.83	116.09 ± 41.10	>0.05
HDL [mg/dl]	56.80 ± 16.12	53.53 ± 13.76	>0.05	56.00 ± 14.75	56.43 ± 17.41	>0.05
TG [mg/dl]	145.21 ± 84.66	121.26 ± 53.59	>0.05	139.21 ± 63.22	142.66 ± 103.62	>0.05
CRP [mg/l]	2.79 ± 4.81	2.57 ± 2.38	>0.05	3.17 ± 5.24	1.96 ± 2.19	>0.05
Creatinine [mg/dl]	0.93 ± 0.15	0.87 ± 0.11	>0.05	0.92 ± 0.14	0.92 ± 0.16	>0.05
Uric acid [mg/dl]	5.39 ± 1.53	5.24 ± 0.79	>0.05	5.33 ± 1.53	5.42 ± 1.19	>0.05

Mg: magnesium, Na: sodium, K: potassium, Ca: calcium, LDL: low-density lipoprotein, HDL: high-density lipoprotein, TG: triglycerides, CRP: *C*-reactive protein, mg/dl, milligram/deciliter; mg/L; milligram/liter.

## 4 Discussion

### 4.1 Rationale for conducting a study looking at the effect of coffee and black tea consumption on sleep parameters

To our knowledge, this is the first study investigating the influence of coffee and black tea intake on sleep architecture using PSG with camera recording. In our study, all patients underwent a single night of audio-video-PSG, a gold standard clinical diagnostic tool for definite SB diagnosis. Previous questionnaire-based studies on bruxism can only suggest a possible bruxism diagnosis, but not confirm it, in accordance with the Lobbezoo et al. consensus [3]. To our knowledge, there are no studies in the available literature investigating the effect of habitual coffee and black tea consumption on sleep parameters. There are however a few studies investigating the effects of coffee consumption right before bedtime [25].

It is generally believed that coffee consumption can worsen sleep parameters and that it possibly favors arousals. However, no recent study supports this hypothesis in habitual drinkers, therefore the aim of this study was to evaluate the effect of habitual coffee and black tea intake on sleep architecture.

### 4.2 The effect of coffee consumption on SB intensity

The patients included in the study declared habitual psychoactive substance use. This is associated with increasing tolerance/resistance for caffeine. Tolerance can develop in about a week of regular use [16,22]. It has previously been suggested that moderate black tea and coffee intake of about 3–4 cups a day, was not associated with any side effects on human health [26]. On the contrary, the consumption of black tea and coffee in these quantities may even be beneficial [27,28]. Its protective effect on the cardiovascular system, liver diseases, diabetes and gastrointestinal disorders has been a subject of interest. Most studies support results suggesting that moderate, and even heavy, coffee consumption, was not associated with a higher cardiovascular risk [[29], [30], [31]]. This is however the first study stating that coffee consumption is correlated with increased sleep bruxism intensity. So far, no study on SB was performed with the use of PSG examination. Up until now, only questionnaires have been used to investigate the usage and effects of stimulative substances on human health. This research indicates the probable influence of coffee consumption on the intensity of teeth grinding. On the other hand, despite increased sleep bruxism parameters, according to our study coffee does not disturb sleep architecture or electrolyte concentrations, including that of magnesium and potassium. This topic requires further investigation in order to establish the possible complications of coffee-induced teeth grinding, including temporomandibular disorders (TMD) and tooth wear [32].

### 4.3 The effect of coffee consumption on sleep architecture

Several authors have correlated coffee and black tea intake with sleep disturbances. However, no study has ever investigated sleep architecture changes and sleep bruxism in relation to coffee and tea consumption. To our best knowledge, the majority of the previous studies based on PSG examination were concentrated on the effect of accidental caffeine intake (administered before bedtime) on sleep architecture. Furthermore, these studies were frequently conducted after sleep deprivation. Moreover, no previous study was ever performed on a large scale [25].

Sleep fragmentation is classified as an interruption in sleep that involves arousals and/or awakenings [33]. Sleep fragmentation impacts neuroendocrine pathways and is a risk factor for various diseases including, among others, cardiovascular, metabolic and mood issues [34,35]. Arousals are linked with excessive somnolence during the day, cognitive performance deficits and mood alterations. In our study, coffee is not significantly associated with an increased arousal-index. Moreover, the index of bruxism associated arousals were similar in coffee drinkers and non-drinkers. This indicates that bruxism has no effect on sleep fragmentation. WASO (wake after sleep onset) was decreased in coffee drinkers, which confirms the beneficial effect of coffee on the continuity of sleep. Thus, habitual drinking of coffee does not appear to promote sleep fragmentation. However, previous studies have shown that accidental drinkers have a lower resistance threshold and are more likely to develop side effects associated with caffeine overdose, including sleep problems [16].

On the other hand, other polysomnographic studies investigating the influence of caffeine on daytime recovery sleep structure, have revealed that caffeine does in fact impact sleep patterns. According to one such study, sleep efficiency, sleep duration, slow-wave sleep and rapid eye movement sleep (REM) were decreased. However, a great limitation of this study was a small study group (n = 24). Moreover, caffeine was given in pills, and PSG was conducted after an entire night of sleep deprivation [36], thus the aim and methodology were different from our study. Salin-Pascual et al. demonstrated that patients suffering from insomnia (n = 6) had a significantly longer sleep latency and less total sleep time in the multiple sleep latency test (MSLT), compared to non-insomniac volunteers after caffeine administration [37]. A questionnaire and diary based survey of 1498 individuals in a French middle-aged working population did not find a significant relationship between total sleep time and daily caffeine intake [38].A survey performed on 515 adults proved an association between poor sleep quality and coffee consumption, however no objective method of sleep estimation was conducted [39].

Several studies conducted in the past on animal models revealed, that caffeine decreased NREM (non-rapid eye movement sleep) and REM sleep stage duration. However, the study was not based on the habitual/regular caffeine influence on sleep architecture, focusing rather on the effects of coffee administered prior to the planned sleep [40].

Numerous studies examining sleep difficulties in military personnel have been published. Consumption of caffeinated products in this study group decreased the participants sleep duration and made it more difficult to fall asleep. It is however worth noting, that these studies were based on self-reported questionnaires without PSG examination [41].

Past studies have shown that a high overall consumption of caffeine, especially in the late afternoon and evening, alters sleep structure, ultimately causing a reduction in both the REM stage of sleep and in total sleep time [42]. Skarupke et al. in a large questionnaire-based German study group of adolescents between the ages of 11–17 years, established that coffee consumption can be associated with complaints of insomnia [43]. The study was conducted on adolescents, which may suggest that the effect of coffee consumption on sleep structure may be age dependent. However, several previous studies presented similar results. A cross-sectional questionnaire study conducted on an Australian study group also suggested that habitual coffee consumption decreases sleep duration, but does not affect its quality [44]. A small PSG-based study investigated the impact of caffeine intake on nighttime sleep. It concluded that sleep architecture did not significantly differ between patients that consumed caffeine, or a placebo or those with withdrawal conditions [45]. Another PSG-based study by Youngberg et al. investigated sleep architecture in habitual moderate (up to 4 cups a day) coffee consumers among primary insomnia patients and good sleepers. No significant differences in sleep parameters were observed between the two study groups [46]. Thus, the result of the Youngberg study is in agreement with our results.

### 4.4 The effect of black tea consumption on sleep architecture

In addition to caffeine, black tea, is also a major theanine source. Due to its similar structure to glutamate and the glutamine neurotransmitter, this amino acid acts antagonistically on glutamate receptors [47]. It has been reported that black tea consumption has a favorable effect on neurocognitive function. The combined caffeine and theanine effect, has been the subject of numerous studies, since both have an influence on neurotransmitter pathways [48]. Caffeine alone is known to have stimulative properties, while theanine has a relaxing effect, promoting calmness and improving cognitive performance [48,49]. Jang et al. reported that small doses of theanine can partially counteract the effects of caffeine on sleep in rats [50]. In our study, black tea drinkers had similar bruxism intensity and sleep architecture as non-drinkers. Therefore, black tea consumption can be recommended even in patients with sleep disorders.

### 4.5 The effect of coffee and black tea on serum concentrations of electrolytes, lipids, CRP and uric acid

Caffeine is a purine alkaloid, a natural xanthine derivative. Purine metabolic pathways in general lead to uric acid production. A Japanese epidemiological study demonstrated a clear inverse relationship between coffee consumption and serum uric acid levels [51]. However, a meta-analysis conducted by Zang et al. showed no significant difference between the highest and the lowest coffee intake categories in terms of the uric acid level [52]. On the other hand Park et al. demonstrated that coffee has a significant lowering effect on serum uric acid [53].Thus, the effect of coffee consumption on the concentration of serum uric acid remains controversial. In our study, coffee consumption did not impact serum uric acid concentration.

Previously, the influence of caffeine on *C*-reactive protein has been considered. In a recent meta-analysis by Moua et al. no statistically significant associations were observed between CRP concentration and coffee consumption in 61,047 participants [54]. In our study, changes in CRP concentrations were also not statistically significant. The result indicates no effect of coffee and tea consumption on the inflammatory process.

It should be kept in mind, that coffee and caffeine vary and are two separate compounds. Coffee is a complex mixture that in addition to caffeine, includes about 1000 other compounds that also impact human health. Trigonelline, one of the main coffee alkaloids along with cafestol, kahweol and chlorogenic acid, is known for its strong anti-inflammatory properties. Other compounds include nicotinic acid, magnesium and potassium [12,14,29]. Potassium and magnesium levels in our study remained in the similar ranges in coffee and black tea drinkers compared to non-drinkers. Thus, our results dispel the myth regarding electrolyte depletion, including magnesium deficiency, in coffee drinkers and are in accordance with previous studies revealing that the diuretic property of caffeine that induces magnesium loss, is compensated by coffee intake itself, due to the high concentration of magnesium in coffee [55].

There are many misconceptions about including coffee and tea as part of a daily balanced diet. However, based on our study results, even patients with sleep disorders can consume coffee on a daily basis, however caution is recommended in sleep bruxers. Nevertheless, excessive daily caffeine intake may lead to physical and psychological dependence, and it should be taken into consideration in choosing healthy dietary choices [56]. Recent findings emerge concerns about disadvantageous influence of COVID-19 pandemic and it should be as well taken into consideration when discussing SB [57,58].

### 4.6 Study strengths

It is remarkable therefore that no polysomnographic study has investigated the role of coffee and black tea consumption on sleep bruxism intensity previously. The strengths of this study include a relatively large study group (n = 106), as well as an additional assessment of electrolyte and lipid panels. All 106 patients underwent an entire night of PSG examination with camera recording, a gold standard tool used in definite SB diagnosis [3]. In addition, PSG examination was performed with the use of video recording that allows to analyze patient's nocturnal behavior, correlate behavior with neuropsychologic parameters and detect epileptiform activity [59].

### 4.7 Study limitations

However, there are also several study limitations. The study group of coffee non-drinkers was small (n = 19). The surveys did not include questions about the type of coffee roasting, caffeine content or information about the method of preparation (e.g. brewed, filtered or boiled coffee), which can influence the results [60]. Patients were divided in four groups regarding coffee and tea drinking habits as mentioned before. However, there are some patients who drink both coffee and tea.

## 5 Conclusions

1. The bruxism episode index is increased in coffee drinkers compared to non-drinkers, thus coffee drinking may be considered a risk factor for definite sleep bruxism.
2. Habitual black tea consumption does not affect sleep bruxism intensity and sleep architecture.
3. Arousal levels, sleep latency and sleep efficiency are similar in coffee drinkers and non-drinkers. Coffee consumption is not related to sleep fragmentation in habitual coffee drinkers.
4. Habitual coffee and tea consumption does not influence CRP, serum uric acid, electrolyte and lipid concentrations in sleep disorder patients.

## Author contribution statement

Weronika Frosztega: conceived and designed the experiments; wrote the paper.

Mieszko Wieckiewicz: conceived and designed the experiments; performed the experiments; wrote the paper.

Helena Martynowicz: conceived and designed the experiments; performed the experiments; analyzed and interpreted the data; contributed reagents, materials, analysis tools or data; wrote the paper.

Dorian Nowacki and Rafal Poreba analyzed and interpreted the data;

Gabriella Lachowicz and Grzegorz Mazur: contributed reagents, materials, analysis tools or data; wrote the paper.

## Funding

Grant number SUBZ. A210.23.040 from the 10.13039/501100009687Wroclaw Medical University, Poland.

## Institutional review board statement

The study was conducted according to the guidelines of the Declaration of Helsinki and approved by the Ethics Committee at Wroclaw Medical University (ID: KB-790/2022).

## Informed consent statement

Informed consent was obtained from all subjects involved in the study.

## Data availability statement

The data that supports the findings of this study is available on request from the corresponding author. The data is not publicly available due to privacy or ethical restrictions.

## Declaration of competing interest

The authors declare that they have no known competing financial interests or personal relationships that could have appeared to influence the work reported in this paper.
