# Supplementary material for: The effect of coffee and black tea consumption on sleep bruxism intensity based on polysomnographic examination
Source: Heliyon. 2023 May 12;9(5):e16212. doi: 10.1016/j.heliyon.2023.e16212 (PMC10205497; doi:10.1016/j.heliyon.2023.e16212)
Supplement: Multimedia component 1 [file mmc1.docx]

**Kwestionariusz dotyczący średniego spożycia kawy i czarnej herbaty w ciągu ostatniego miesiąca**
 **(Questionnaire regarding average coffee and black tea consumption per day in last month)**

Proszę zaznaczyć średnią ilość wypijanych napojów dziennie w ciągu ostatniego miesiąca w filiżankach.
Please give average amounts of beverages per day in last month in cups.

1. Dzienna ilość wypijanych filiżanek kawy 0/1/2/3/4/5/6/7/8/1/9/10

(1. Daily cups of coffee 0/1/2/3/4/5/6/7/8/9/10)

2. Dzienna ilość wypijanych filiżanek czarnej herbaty 0/1/2/3/4/5/6/7/8/9/10

(2. Daily cups of black tea 0/1/2/3/4/5/6/7/8/9/10)

Participant ID:
